# Environmental Factors and WASH Practices in the Perinatal Period in Cambodia: Implications for Newborn Health

**DOI:** 10.3390/ijerph120302392

**Published:** 2015-02-23

**Authors:** Alessandra N. Bazzano, Richard A. Oberhelman, Kaitlin Storck Potts, Anastasia Gordon, Chivorn Var

**Affiliations:** 1 Department of Global Community Health and Behavioral Sciences, Tulane University School of Public Health and Tropical Medicine, 1440 Canal St., New Orleans, LA 70112, USA; E-Mails: oberhel@tulane.edu (R.A.O.); kstorck@tulane.edu (K.S.P.); agordon8@tulane.edu (A.G.); 2 National Institute of Public Health, #2 Kim Y Sung Blvd, Tuol Kork, Phnom Penh P.O. Box 1300, Cambodia; E-Mail: chivorn@rhac.org.kh

**Keywords:** hygiene, WASH, newborn mortality, health facility strengthening, neonatal infection, structured observation, qualitative research

## Abstract

Infection contributes to a significant proportion of neonatal death and disability worldwide, with the major burden occurring in the first week of life. Environmental conditions and gaps in water, sanitation and hygiene (WASH) practices may contribute to the risk of infection, particularly in settings where health centers are expanding to meet the growing demand for skilled care at birth and homes do not have adequate access to water and sanitation. A qualitative approach was used to understand the environmental context for infection prevention and control (IPC) and WASH associated behaviors in health centers where women give birth, and in homes of newborns, in a rural Cambodian province. Structured observations and focus group discussions revealed important gaps in optimal practices, and both structural and social barriers to maintaining IPC during delivery and post-partum. Solutions are available to address the issues identified, and tackling these could result in marked environmental improvement for quality of care and neonatal outcomes. Water, sanitation and hygiene in home and health center environments are likely to be important contributors to health and should be addressed in strategies to improve neonatal survival.

## 1. Introduction

Over the last few decades, remarkable improvement in newborn survival has occurred, albeit at a slow pace. It is estimated that between 1990 and 2012, mortality among babies less than 28 days old globally decreased by 2% each year [1,2]. While encouraging, this progress must be sustained and accelerated, particularly as newborn mortality accounts for 44% of all deaths of children under 5 years of age [1]. Without progress in newborn survival, child mortality will not improve. In Cambodia, where maternal mortality has decreased markedly over the last decade due to a concerted effort to improve service delivery [3,4,5], there has been substantially less progress in reducing newborn mortality [6,7,8]. A renewed effort has been made to understand and address the factors underlying this situation. Important causes of newborn mortality in Cambodia are preterm birth, birth asphyxia, sepsis and pneumonia, congenital malformations and tetanus [9].

Globally, an estimated 35% of newborn mortality can be attributed to infection, including: sepsis, meningitis and pneumonia [10], and morbidities related to infection account for approximately 3% of all disability adjusted life years (DALY’s) [11,12]. Verbal autopsy data collected in Svay Rieng, Cambodia in 2009 indicated that bacterial sepsis was the likely cause of death in 40% of neonatal deaths, by far the largest category (exceeding the next highest cause by 17%) [13].

Infection prevention and control (IPC) and clean care practices surrounding delivery and the post-partum period have been identified as essential to the prevention of neonatal mortality [14,15,16,17], but gaps remain in rural delivery and newborn care environments in low and middle-income countries. Three quarters of newborn mortality occurs in the early postnatal period, during the first seven days of life [18]. Given the high burden of disease, assessing environmental and IPC practices and exposures during this period is an important step.

This formative research study aimed to explore the environmental conditions that surround the perinatal period and may impact on newborn health. This dimension of the care environment is comprised of households, communities and health centers (HCs) where care of newborns occurs. The results of the study were integrated into a planned intervention to improve newborn health.

## 2. Experimental Section

The study employed a number of qualitative methods including structured observation, in-depth interviews and focus group discussions to investigate environmental conditions and WASH practices in two related settings: HCs where women give birth and home environments, in Takeo Province, Cambodia. Takeo province is a rural agricultural region and is located in the south of Cambodia, near the border with Vietnam. During February–April 2014, data were collected in HC catchment areas in the province. The population that comprised the HC catchment areas included those who used the HC for delivery and for whom it was the nearest primary care facility. Table 1 indicates the data collection methodology.

**Table 1:** Data collection methods and participants.

Methods	Data Collected	Participants	Location
Observation, photos, video	Hygiene and IPC practices in health centers and related equipment and supplies	10 Health centers	Health center
	Newborn care practices related to WASH	4 Newborns (less than 29 days old) and their mothers at home	Home
Semi-structured interviews	Hand washing Diapering Water and Sanitation Bathing	27 Mothers/Caregivers; 16 village health support group volunteers	Health center
Focus group discussions	Hygiene and IPC practices in health centers Newborn care practices	2 Focus groups	Health center

For the HC environment, structured observations using a checklist (derived from validated tools) were conducted at 10 HCs. This information complemented two focus group discussions conducted with midwives and staff in HCs on practices, and collection of photo and video documentation of the HC environment and equipment used there during the perinatal period.

The home environment and practices were assessed through semi-structured, in-depth interviews with mothers or primary caretakers. The interview results were augmented by four home observation sessions, which recorded hygiene behaviors and resources in the homes of newborns. Village health support group (VHSG) volunteers were also interviewed to understand community perspectives.

The structured observation data was aggregated and summary statistics were determined to assess the state of hygiene infrastructure and resources in the HCs and at home. Thematic analysis of the interviews and focus groups was employed to determine hygiene practices and behaviors of HC staff and primary caretakers, and to determine other barriers to achieving optimal environments for newborns. Photos and videos were compiled and categorized to triangulate data gathered on practices (e.g., newborn bathing at home) and environmental conditions (e.g., condition of delivery and post-delivery room and equipment). Table 2 illustrates the items included in the HC and home environment checklists.

**Table 2:** Structured observation checklist items for the health center and home environments.

Domain	Features Checked
**Health Center Environment**	
Infrastructure	Electricity
	Backup electricity
	Designated rooms for delivery and post-delivery care
	Sufficient lighting for health care activities
Hand hygiene	Hand washing points
	Soap present Clean towels for drying present Near to areas where health care carried out Near to latrines
Routine cleaning	Condition of:
	Floors Operating tables Surfaces which mother or newborn may contact
	Availability of cleaning supplies
	Availability of cleaning equipment
Sanitation	Latrines
	Adequate Clean latrines Accessible for all users
	Sharps disposal
	Waste disposal
Water	Indoor running water
	Outdoor running water
	Appearance of water
Sterilization	Sterilizing equipment
	Electric autoclave
Health care equipment and supplies	Sterile/non-sterile gloves
	Plastic sheeting for delivery
	Clean linens for delivery and post-partum care
	Clean cord care items
**Home Environment**	
Sanitation	Toilet present
Animals	Presence of animals
Hand hygiene	Hand washing station at toilet
	Water at hand washing station
	Soap at hand washing station
Water	Source of household water
	Storage of household water

Criteria for inclusion of HCs in purposive sampling included the following: geographic location, number of HC deliveries conducted each month, location relative to roads, socioeconomic status and urban/rural designation. Study participants included midwives, primary caregivers of children under 2 years of age (including mothers, grandmothers, and fathers), as well as Village Health Support Group volunteers (VHSG). Midwives participated in two Focus Group Discussions (FGDs) ranging from 4–6 participants per group. They were recruited on a convenience basis from HCs selected for structured observation, and were not provided incentives for participation. There was one moderator and one note taker per group. The criteria used to identify caregiver participants included: urban/rural residence, socioeconomic status, age, and parity (for mothers).

Data were collected from two FGD with midwives, 27 semistructured interviews with caregivers, 16 interviews with VHSG and observation sessions (lasting between 1–4 h), of which ten were conducted in the HC and four with recently delivered women and their newborns at home. Additionally, photos and videos were taken with explicit permission. All interviews and FGD were audio recorded and notes were taken by researchers and assistants with multiple transcribers for cross-checking.

Thematic analysis was used by one experienced researcher and one assistant to identify the most pertinent themes from the data. The researchers used a predetermined coding scheme, based on the interview and FGD guides, as well as the structured observation tool items. NVivo software for qualitative analysis was used for coding and managing data through the analysis phase.

The study protocol was reviewed and approved by the Tulane University Biomedical IRB and the Cambodian National Ethics Committee for Health Research. Informed oral consent was obtained for observations and informed written consent was obtained from all focus group and interview participants, as well as for use of photos and videos.

## 3. Results

### 3.1. Immediate Newborn Care Environment: Structured Observations of Health Centers

Structured observations of 10 HCs revealed barriers to optimal environmental sanitation and IPC that could potentially impact health of newborns and their mothers.

#### 3.1.1. Infrastructure

##### Electricity

All of the HCs had electricity, although one was experiencing an outage without backup power during the observation session so was recorded as not having electricity. Only five of the 10 HCs had a backup generator or other secondary source of electricity.

##### Designated Delivery and Post-Delivery Rooms

All of the HCs had designated delivery and post-delivery rooms, though many had a limited number of beds. Excluding the referral hospital, the HCs had one to three delivery beds and two to four post-delivery beds. In some cases, post-delivery rooms were being used for storage or office space, or a multi-purpose room was used both for examining general clinic patients and as a pre- or post-delivery room when necessary.

##### Sufficient Lighting for Healthcare Activities

All 10 HCs had a well-lit delivery room, however HCs were visited exclusively during the day for observations and data collection. Whether sufficient lighting is available without the aid of natural light from windows was not assessed.

#### 3.1.2. Hand Hygiene

Nine of the rural HCs did not have adequate hand washing facilities in all areas where healthcare took place. The referral hospital had alcohol hand sanitizer located throughout the maternity section. All of the HCs had indoor running water for hand washing, but only three HCs had soap, or a suitable alternative, present at all hand washing points and only one HC had clean towels available at the hand washing stations. Air-drying of hands is not practiced in the area and observers noted dirty towels were used for hand drying. Additionally, only four of the 10 HCs had hand washing points near the toilets. All 10 HCs had sinks in the delivery rooms, but only four had a hand sanitizer or disinfectant in the delivery rooms.

#### 3.1.3. Routine Cleaning

##### Condition of Floors, Operating Tables, Surfaces for Mother and Newborn

Environmental condition in many HCs was sub-optimal based on the observation of data collectors and as recorded in photographs. In particular, there were areas in delivery and post-delivery rooms with surface dirt, cobwebs and dust, along with equipment (e.g., ambu bags) that appeared not well maintained. Per the observation visits, three of the 10 HCs did not have an adequately clean delivery room and only half had clean delivery beds. Six of the HCs had plastic sheets for delivery beds but the condition of those was varied and last cleaning of those could not be assessed. Seven of the 10 HCs did not have clean clothes or towels to dry the baby, and did not have clean newborn scales.

##### Availability of Cleaning Supplies and Equipment

Two HCs did not have a visible detergent product for cleaning the delivery room, and eight of the HCs did not have bleach. The majority (seven) had no disinfectant for the delivery room environment. Most HCs did have brooms and mops available to clean the delivery rooms, though these were occasionally stored in the same room as the toilet for staff.

#### 3.1.4. Sanitation

##### Latrines

Though many of the HCs have toilets, they are often not clean or accessible to all users. Three of the 10 HCs did not have adequate toilet facilities and at only four HCs were the latrines accessible to all users—the latrine was often outside the facility far from the delivery and post-delivery area. The majority of toilets were rated as not adequately clean by observers and only the referral hospital had clean toilets without smell.

##### Sharps Disposal

All 10 HCs had a sharps disposal. These generally consisted of a cardboard box with appropriate insertion point for sharps. In some cases, the sharps disposal was observed to be overflowing or not adequately sealed. Midwives reported the sharps disposal box were eventually either burned or buried.

#### 3.1.5. Water

All 10 HCs had indoor running water available and 8 also had outdoor taps for use. The indoor and outdoor taps were in working order during the observation sessions. Half of the HCs did not have an alternative source of water in case of emergency need. In addition, four HCs did not have an adequate supply of well water. Observers noted that stored water was contaminated at many of the HCs with visible debris or a film on the surface with a murky or cloudy color. This was further documented in photos. Outside containers of stored water were often without covers to prevent contamination. The water was from improved source and was not additionally treated at the HCs.

#### 3.1.6. Sterilization

Though all 10 HCs had an autoclave to sterilize instruments, only seven were clearly in working order. Many of the steam sterilizers were dusty or had visible cobwebs.

#### 3.1.7. Health Care Equipment and Supplies

The majority of the HCs did have either sterile or non-sterile disposable gloves, though midwives reported frequent shortages. Only four of the HCs frequently launder linens with soap and water. Most HCs do not have laundering facilities. Seven HCs had clean and sterile unused umbilical clamps, and only five had clean bandages for the umbilical cord. Observation items recorded via the HC checklists are summarized in Table 3.

**Table 3:** Summary scores from health center observations.

Domain	Number of Health Centers with Feature	Number of Health Centers without Feature (Missing)
**Infrastructure**		
Electricity	9	1
Backup electricity	5	5
Delivery room	10	0
Post-delivery room	10	0
Sufficient lighting in delivery room	10	0
**Hand Hygiene**		
Adequate hand washing points	1	9
Soap present	3	7
Clean towels present for drying hands	1	9
Near toilets	4	6
**Routine Cleaning**		
Delivery room clean	7	3
Post-delivery room clean	6	4
Newborn scale is clean	3	7
Delivery bed is clean	5	5
Detergent present for cleaning delivery room	7	2 (1)
Disinfectant present for delivery room	1	7 (2)
Brooms or mops present for cleaning	9	0 (1)
**Sanitation**		
Adequate toilets	6	3 (1)
Toilets are clean	1	9
Toilets are accessible to all users	4	6
There is a sharps disposal present	10	0
**Water**		
Indoor running water	10	0
Outdoor running water	8	2
**Sterilization**		
Electric autoclave	10	0
**Health Care Equipment and Supplies**		
Sterile or non-sterile gloves	9	0 (1)
Plastic sheeting for delivery	6	2 (2)
Clean towels or cloths for drying baby	3	7
Clean, sterile, unused umbilical clamps	7	2 (1)

**Figure 1:**
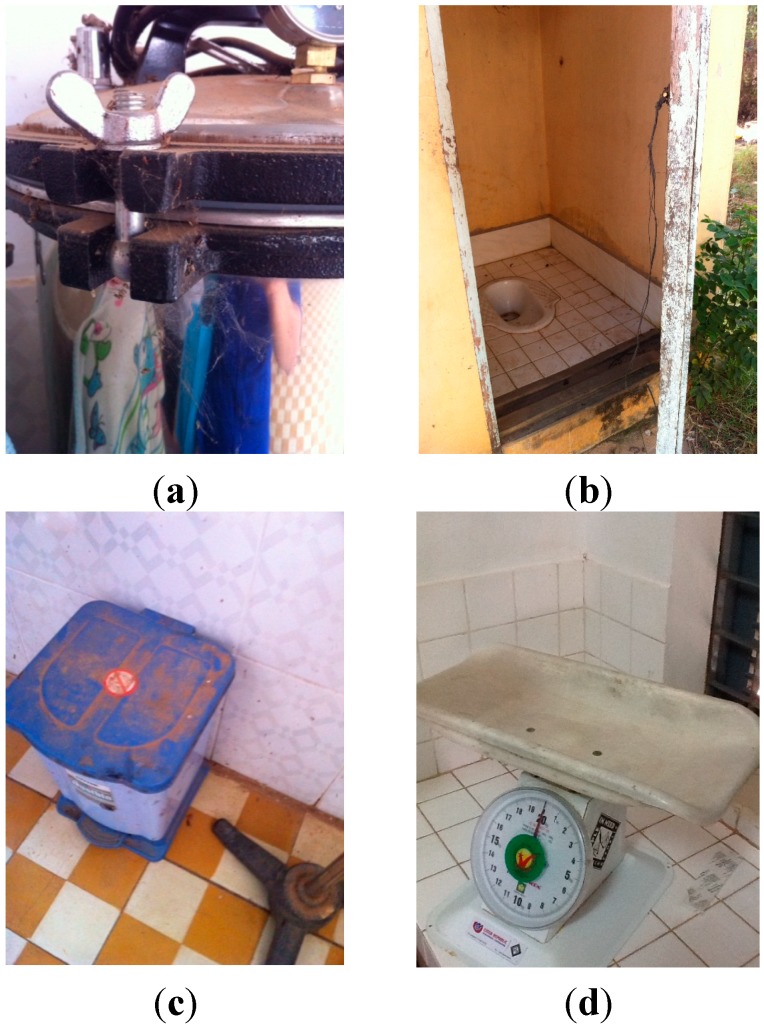
(**a**–**d**) Photos of health centers.

Some items could not be assessed at all HCs due to observation constraints. These are recorded as missing. Some of the conditions of observed HCs can be seen in the images in Figure 1. These photos illustrate (a) cobwebs on equipment; (b) patient latrines, which are far from the delivery room and not sufficiently clean; (c) dust found on floors and equipment; (d) newborn weighing scale.

### 3.2. Immediate Newborn Care Environment: Focus Group Discussions with Health Center Staff

During focus group discussions and follow up at HCs, the lack of janitorial staff was mentioned as a barrier to regular cleaning and sanitation in the facility. Midwives are expected to ensure the cleaning of the delivery area after each delivery.

The midwife who handles the delivery is responsible for all hygiene and cleaning after that delivery (the laundering of the white cloths (for drying baby), asking the family to clean the delivery room table and floor after the delivery, throwing away used supplies such as needles, sutures.

*(Primary midwife, 24 years old)*

At one HC, midwives described having a cleaner who was expected to come once a day to clean the entire facility, but it was noted that the cleaner sometimes did not come to do that work.

The hygiene is sometimes not good enough because the cleaner does not come often.

*(Primary midwife, 28 years old)*

A typical practice reported by participants in focus groups was to ask the family of the pregnant woman to clean the delivery area after the birth. Midwives described it this way:

After the delivery we need to wash some items with soap and water then we will sterilize it later. So that is the scissors, forceps, catheter. Other items will be thrown away. We give the disposables such as the needles, sutures and other things to the family along with the placenta for them to bury somewhere.

*(Primary midwife, 24 years old)*

We ask the family to sweep, use the broom and also to use dishwashing soap to clean the floor if the floor was tainted by amniotic water. It’s not usual that there is amniotic water on the floor. We use a bucket to catch amniotic water but it sometimes spills over onto the floor.

*(Primary midwife, 46 years old)*

The same midwife noted that she had received her training on IPC a long time ago, and now had forgotten some of it, indicating that she would like to receive refresher training. Another midwife, who finished her studies more recently, described her training this way:

I did have training on infection prevention and control at midwifery training and it was mainly about how to prevent myself from getting HIV from a patient using the protective glasses, long gloves but also now we do HIV testing and refer those women who have HIV to delivery elsewhere.

*(Primary midwife, 24 years old)*

Regarding hand hygiene practices, she reported the following:

Yes we do wash our hands with bar soap and then apply the gel before putting our gloves on. If we are rushing we just use 2 pairs of gloves. We use the same gloves from start to finish with the mother and the newborn. We don’t change the gloves until we are completely finished with the mom and the baby, including the weighing of the baby.

During discussions, midwives stated that there were sometimes shortages of gloves used during deliveries. On these occasions, if a sufficient number of gloves were not available, HC staff would purchase them at a local market.

### 3.3. Post Partum Environment: The Home and Community

#### 3.3.1. Home Observation and Interviews with Mothers and Primary Caregivers

Structured observations were conducted at the homes of four newborns, and unstructured observations were carried out opportunistically with mothers participating in semi-structured interviews. A summary of results from the four structured home observations can be seen in Table 4.

**Table 4:** Summary of structured home observations: environment and newborn care practices.

Environment	Number of Homes Observed with Feature or Behavior (*n* = 4)
**Sanitation**	
Toilet	2
**Animals**	
Presence of animals	2
**Hand Hygiene**	
Hand washing station at toilet	0
Water at hand washing station	0
Soap at hand washing station	0
**Source of Drinking Water**	
Well water	2
Pond water	1
Bottled water (purchased)	1
**Behaviors and Newborn Care**	
Washed hands before touching newborn	0
Changed diaper when soiled	3
Bathed newborn	3

During interviews with mothers and caregivers, some basic WASH related practices were explored. Hand washing practice varied greatly; with some participants stating that they did not wash their hands and others who stated it was important for newborn health. No family member was observed washing his or her hands before touching the newborn during the home observations. Mothers described their hand washing habits in interviews.

We never wash hand before touching the baby, because every time we hold the baby is for giving bath, so we don’t need to wash our hands, for me during the baby was under 1 month old, I did nothing, I rest on the bed all the time and I didn’t touch anything. I wash my hands every time I use the toilet.

*(Mother, 23 years old)*

We mostly don’t wash hands before touching the baby, but I wash my hands after using the bathroom.

*(Mother, 31 years old)*

My mother always washed her hands before taking care (of the newborn). I only wash my hands during my regular shower, 3 times a day.

*(Mother, 27 years old)*

I never paid any attention to hand washing, there’s not enough time, or I forget. So when I finish weaving I just take the baby (without washing my hands). I don’t have a hand washing habit. I was told by the midwife to wash hands.

*(Mother, 38 years old)*

Many caregivers did describe washing hands and had good knowledge of why it was important. But sometimes it simply doesn’t happen, as this participant explained:

I have heard advice about washing hands regularly and I practice that. Especially before eating anything. And I also heard that after toilet you should wash hands. I know that is to protect from germs in the stool that you might touch. These germs from the stool can be anywhere in the area of the latrine, they can be everywhere we cannot see. But sometimes we do not wash our hands before touching the baby. Yes that happens.

*(Grandmother, 55 years old)*

**Figure 2:**
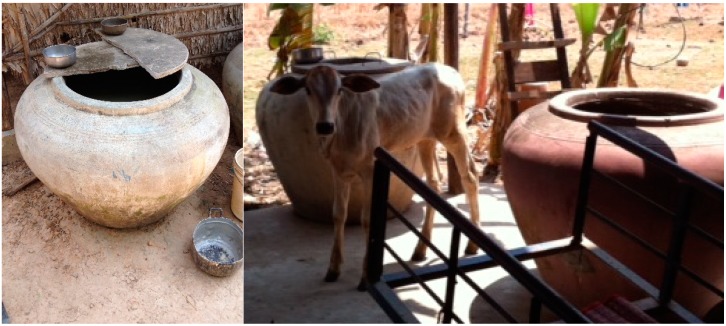
Water storage in the home environment.

Interviews also included the topic of infrastructure and resources related to hygiene behaviors in the home. Many of the observed families did not have toilets. Two of the families used a shared neighbor’s toilet and the other two practiced open defecation. None of the households had hand washing stations near the toilets, when toilets were available. Water sources reported by participants included well water, pond water (typically used in the rainy season), and water purchased for consumption. Three of the 4 households observed used well water as their primary source of water. However, one of the mothers who reported well water also indicated that she purchased bottled water for cooking and drinking. The team observed some wells which were working and some which were not functional—participants confirmed that some wells were either broken or were not used because of water that was “smelly” or had a “bad taste.” The remaining observed household fetched water from a nearby pond. Storage of water was typically in large earthen containers situated around the outside of the home. These wide-mouthed containers were often not covered, and those that were covered were not sealed well. Items used to retrieve water from storage were left out in the open and during home visits multiple persons were routinely observed placing potentially contaminated items (such as hands, cups and ladles) into the stored water containers. Storage of water is pictured in Figure 2 below.

A community leader described water sources in one village:

There are 142 total households and most get water from shared well. Some wells have clear water and use for cooking/drinking, other wells are not clear (and only used) for laundry. Only 2–3 families have pond in dry season. Rainy season have more ponds and they use that water for washing clothes, bathing or boil for drinking.

*(Village Chief, 72 years old, male)*

One participant described water and hygiene issues in the community this way:

Mothers leave babies with grandmas to go to the field and the grandmas give baby unclean water with sugar (making them ill). If the mother doesn’t clean the house, the baby will get a lot of sickness, and get into a bad condition because of the dust in the house. Families in not so good condition store water in huge clay jars outside the house, but families in good condition use machine to filter water. Some also boil water to avoid diarrhea.

*(Village Health Volunteer, 46 years old, female)*

**Figure 3:**
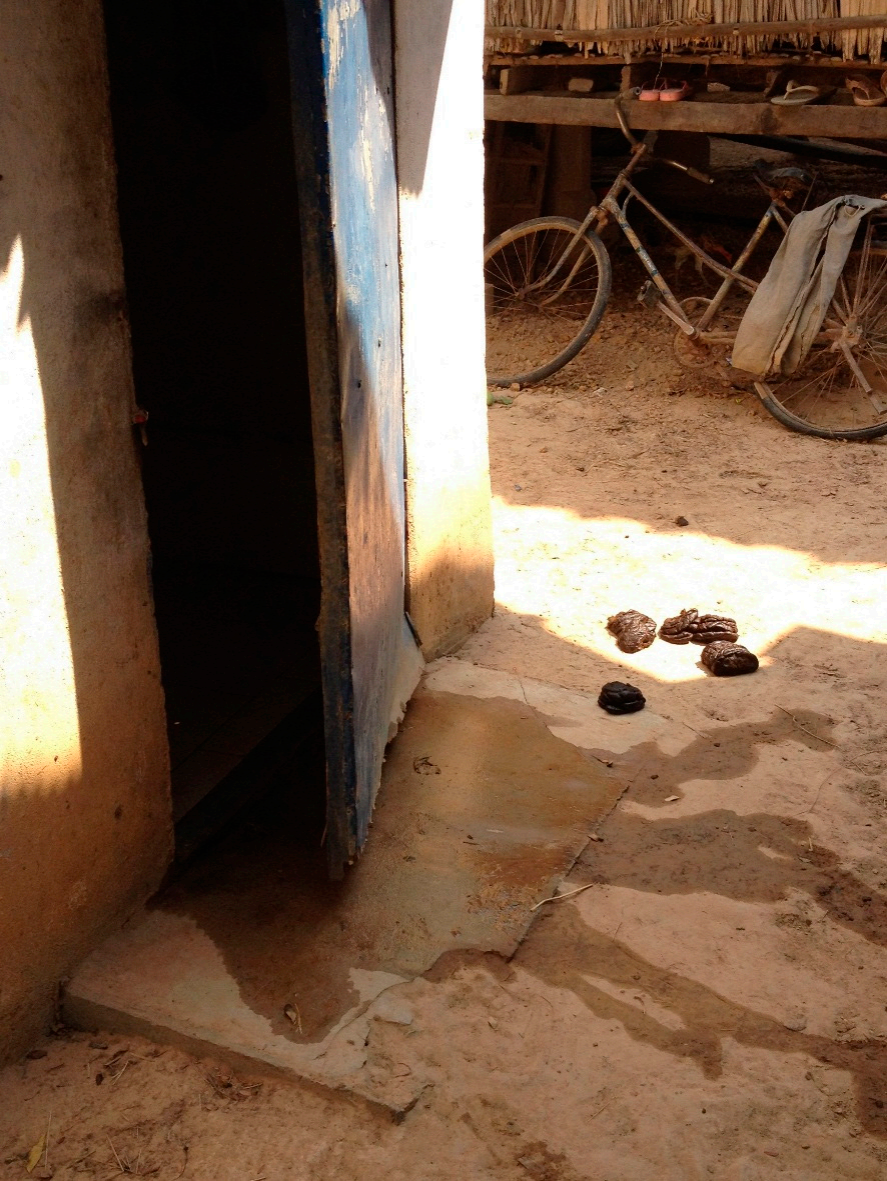
Animal feces around the home setting.

In addition to inadequate water and sanitation facilities, researchers sometimes observed animals such as cows, chickens and dogs, and animal feces, present in close proximity to the home area where care giving of the newborn took place (see Figure 3 below).

**Figure 4:**
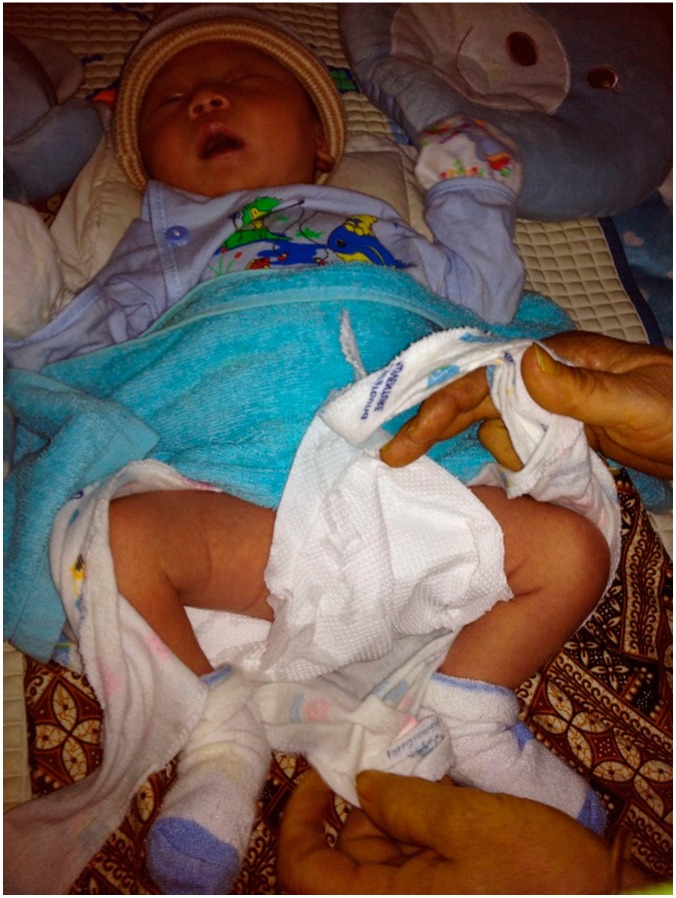
Diapering of a newborn.

Other hygiene related factors that were explored through interviews included bathing, care of the umbilical cord, and diapering. Diapers described to and observed by the research team consisted of a paper towel interior with a cloth wrapped around the exterior of the newborn’s body. All mothers were observed or reported changing the newborn’s diaper when soiled. Disposal location of the soiled paper towel interior varied from a trash bag to adding it to a pile of refuse near the home. Figure 4 illustrates a typically diapered newborn infant.

One mother described the typical practice of washing diapers for a newborn baby:

We have 12 (cloth) diapers and washed 2 times a day all with well water, (we use) cold water in well container, powder soap, my husband does it. Paper towel from the diapers is buried or burned.

*(Mother, 22 years old)*

Additional newborn care behaviors were noted during the home observations. While some mothers were observed bathing the newborn, none used soap to do so. All newborns were clothed or wrapped, though one mother wrapped the newborn in the same cloth she used to dry the baby after bathing, leaving the baby wrapped in a damp cloth with its chest exposed.

During interviews the research team also explored issues related to safe water and the use of infant formula, water, and washing of feeding bottles. Caregivers described use of breast milk substitutes that require water for preparation and providing water to babies.

During that 1st month, the baby only received condensed milk mixed with water.

*(Father, 30 years old)*

I gave (baby) water with spoon and cup, boiled water, each time after breastfeeding.

*(Mother, 26 years old)*

**Figure 5:**
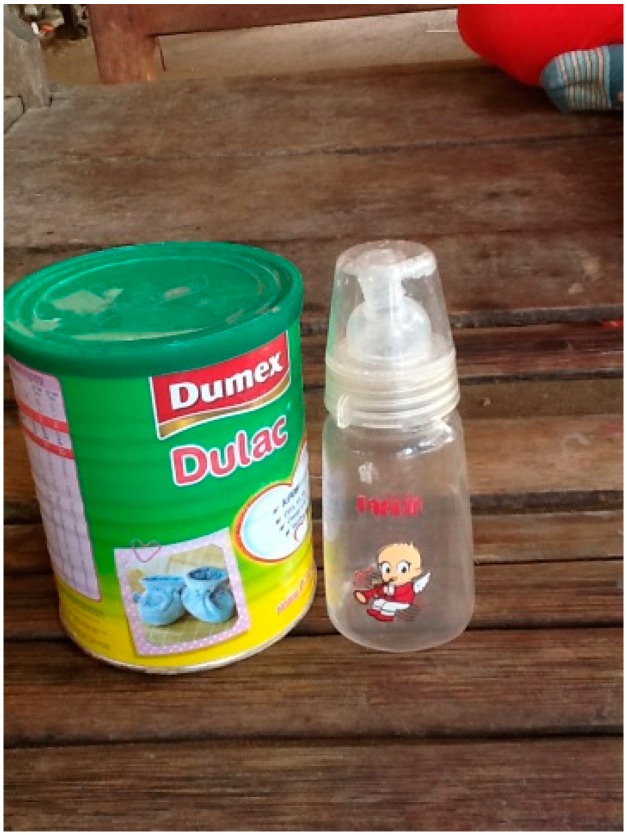
Feeding bottle.

On a couple of occasions, caregivers reported that they did not provide water to newborns but water in feeding bottles was observed in the home near the areas where care-giving occurred. Figure 5 illustrates a feeding bottle used by a participant.

## 4. Discussion

### 4.1. Findings and Discussion

The data presented highlight gaps in WASH practices and underlying environmental barriers to optimal hygiene within the settings in which newborn care takes place in a rural Cambodian province. While these findings are not surprising giving the severe resource challenges and lack of access in low income settings, they should be recognized and incorporated into strategies aimed at improving newborn and maternal health at the community level.

The World Health Organization estimates that on average in Cambodia 61% of the population have access to an improved drinking water source and 29% of the population have access to improved sanitation, with disparities between urban and rural areas [19]. The findings from this study indicated similar levels of access to improved water and latrines, which are likely to be important to reducing transmission of infections. Further, the results illustrated issues with water storage at HCs despite access to improved water sources. Inappropriate storage and use practices at the HCs along with inadequate sanitation are important barriers to a safe environment and should be addressed for improved newborn health outcomes. Home environments often also included the presence of animals and animal feces near to areas where newborn care is carried out, another potential source of enteric infections [20,21].

Although we did not observe hand washing practices in great detail due to time and resource constraints [22,23], the observations, checklists, and interviews indicated that hand washing practice at the household level was suboptimal in the study area. Few studies have directly examined the relationship between hand washing behavior and impact on newborn mortality [24,25], but improving hand washing behavior in both home and facility settings is likely to be an important step in improving IPC for newborns.

Our findings on IPC and hygiene practices in HCs echo previous research in Cambodia by Ith and colleagues [26], and we concur with the conclusion of that study. Gaps in optimal practices are directly related to the lack of a supportive working environment for skilled birth attendants and lack of resources in HCs. HC midwives ought not to be tasked with cleaning delivery and post-delivery rooms on their own, nor should they have no choice but to enlist the help of patient’s families in these tasks, however the lack of financial resources has resulted in few if any janitorial staff for the HCs. Hiring and training sanitation workers for all HCs (as was done in two of the HCs visited) would be an important step in supporting midwives in providing quality perinatal care.

To our knowledge, this is one of few formative research studies on newborn care practices that explicitly includes qualitative and observational exploration of environmental conditions and WASH in both the facility and home environment during the perinatal period. Hygiene practices during home births and research on improving clean care at delivery are acknowledged as important to ensuring newborn survival and thus much formative research has focused on these topics [27,28,29,30]. However, less attention has been given to general environmental conditions and WASH practices that form the context of delivery and post-partum care. A major review of community-based interventions for improving neonatal health outcomes in developing countries did not include any interventions that specifically addressed WASH practices in the home or IPC in the facility setting [31]. As this study was limited by scope and is not intended to be generalizable due to the qualitative methodology, further research on a larger scale may be warranted to understand the potential for impact.

Recent studies have been conducted on individual water and sanitation issues related to child health. Given the need for increased access to improved sanitation facilities, research has addressed provision of latrines as one intervention. The results of a recent study suggested that changing the structural environment might not be enough to combat infections. A cluster-randomized trial, conducted in rural India, found that increased access to safe latrines alone did not result in improvements in child infection and malnutrition [32]. Although the researchers did not assess the impact of increased latrines on neonatal mortality specifically, the results likely apply to this vulnerable sub-group.

As hand washing practice by health providers has been studied extensively and is positively linked to improved health outcomes, a focus on this topic may be warranted. Interventions to improve hand washing behaviors in home and community settings are also important. In one recent study of a potentially scalable behavioral intervention, communities were found to have sustained improved hand washing practices even 5 years after they had received the intervention [33]. More recently, the “Super Amma” campaign in rural India found increased hand washing with soap after the intervention targeted emotional drivers of behavior [34], which could be more important in the household and community setting. Such results highlight the potential for success of interventions that focus on motivational factors beyond simple education. Motivational factors may include behavioral drivers like mother’s desires to nurture their children, social norms, and disgust of dirtiness [35]. A recent systematic review of hand hygiene intervention strategies also discussed the need to address determinants beyond knowledge, awareness, action control, and facilitation in order to change hand hygiene behavior [36]. These strategies could be used in the context of any intervention on newborn health in order to increase hand washing and hygiene practices around newborn care.

### 4.2. Limitations

This research was limited in scope in several ways. The results identified may not be applied to geographic regions beyond the area studied. The home observation sessions were limited in number and therefore are not able to depict the range of newborn home care environments in the region, but offer illustrations of potential environmental situations newborns face when they leave the HC. The HC observation sessions took place over a limited timeframe, and were not repeated, and therefore may not be representative of the usual state of the HC environment.

## 5. Conclusions

Environmental conditions and WASH related barriers in the study area are an important point of reference for understanding health and may be a factor impacting on perinatal and neonatal outcomes. Potentially scalable solutions to address these issues are available and should be considered where interventions designed to increase newborn survival are implemented. Provision of hand washing materials and behavior change related to hand washing is an important potential strategy. Similarly, HCs must be strengthened to have access to essential equipment, supplies, refresher IPC training and janitorial personnel to ensure a sanitary environment for mothers and newborns.
